# Perceived Delay in Healthcare-seeking for Episodes of Serious Illness and Its Implications for Safe Motherhood Interventions in Rural Bangladesh

**Published:** 2006-12

**Authors:** J. Killewo, I. Anwar, I. Bashir, M. Yunus, J. Chakraborty

**Affiliations:** ^1^ Reproductive Health Programme (present address: Department of Epidemiology and Biostatistics, Muhimbili University College of Health Sciences, Medical Centre, PO Box 65015, Dar es Salaam, Tanzania); ^2^ Matlab Health Research Centre; ^3^ Matlab Community Health Research Branch, ICDDR,B, GPO Box 128, Dhaka 1000, Bangladesh

**Keywords:** Healthcare, Emergency obstetric care, Behaviours, Maternal mortality, Interventions, Safe motherhood, Cross-sectional studies, Bangladesh

## Abstract

Delay in accessing emergency obstetric-care facilities during life-threatening obstetric complications is a significant determinant of high maternal mortality in developing countries. To examine the factors associated with delays in seeking care for episodes of serious illness and their possible implications for safe motherhood interventions in rural Bangladesh, a cross-sectional study was initiated in Matlab sub-district on the perceptions of household heads regarding delays in seeking care for episodes of serious illness among household members. Of 2,177 households in the study, 881 (40.5%) reported at least one household member who experienced an illness perceived to be serious enough to warrant care-seeking either from health facilities or from providers. Of these, 775 (88.0%) actually visited some providers for treatment, of whom 79.1% used transport. Overall, 69.3% perceived a delay in deciding to seek care, while 12.1% and 24.6% perceived a delay in accessing transport and in reaching the provider respectively. The median time required to make a decision to seek care was 72 minutes, while the same was 10 minutes to get transport and 80 minutes to reach a facility or a provider. Time to decide to seek care was shortest for pregnancy-related conditions and longest for illnesses classified as chronic, while time to reach a facility was longest for pregnancy-related illnesses and shortest for illnesses classified as acute. However, the perceived delay in seeking care did not differ significantly across socioeconomic levels or gender categories but differed significantly between those seeking care from informal providers compared to formal providers. Reasons for the delay included waiting time for results of informal treatment, inability to judge the graveness of disease, and lack of money. For pregnancy-related morbidities, 45% reported ‘inability to judge the graveness of the situation’ as a reason for delay in making decision. After controlling for possible confounders in multivariate analysis, type of illness and facility visited were the strongest determinants of delay in making decision to seek care. To reduce delays in making decision to seek care in rural Bangladesh, safe motherhood interventions should intensify behaviour change-communication efforts to educate communities to recognize pregnancy-danger signs for which a prompt action must be taken to save life. This strategy should be combined with efforts to train community-based skilled birth attendants, upgrading public facilities to provide emergency obstetric care, introduce voucher schemes to improve access by the poorest of the poor, and improve the quality of care at all levels.

## INTRODUCTION

One of the contributing factors to high maternal mortality in developing countries is the delay in accessing emergency obstetric-care (EmOC) facilities during life-threatening complications. This delay has been well-described as consisting of three levels: delay in making decision to seek care, delay in arrival at a health facility, and delay in receiving adequate treatment, which have been named first, second, and third delay respectively (1). There is now no doubt that addressing obstetric complications is most crucial in the reduction of maternal mortality (2). However, in Bangladesh and many other developing countries, little has been done to use the three-delay model to improve access to EmOC services because its facets are complex and inter-related and involve various factors, particularly those relating to promptness in making final decisions to seek care. Several attempts have been made by countries to reduce delay in accessing care for obstetric emergencies, such as increasing geographical coverage of facilities, providing transport, and improving quality of care (3–6), but these are locality-specific and are difficult to replicate and sustain in other settings.

Bangladesh is one of the poorest countries in the world with high maternal mortality (322 per 100,000 livebirths) (7). The country has taken up the issues of the safe motherhood initiative and the recommendations of the International Conference on Population and Development as a priority (8). Bangladesh has previously adopted a strategy of upgrading peripheral public-health facilities to provide comprehensive EmOC. The programme started in 1994 with the target to provide quality comprehensive essential obstetric care (EOC) services from all 13 medical college hospitals, 59 district hospitals, 64 of 90 Maternal and Child Welfare Centres (MCWCs), and selected (132 of 403) sub-district-level rural Upazila Health Complexes (UHCs). Progress so far is slow; to date, all 13 public-sector medical colleges, 59 district hospitals, and 64 targeted MCWCs are providing comprehensive EOC services, but only 75–80 of the 132 targeted UHCs are functioning as comprehensive EOC facilities. Use of the upgraded facilities remains low (9–14). Barriers to the use of facility-based services as reported in Bangladesh and elsewhere are social, e.g. restricted mobility of women, cultural, e.g. preference of home-delivery, and economic, e.g. widespread poverty. To overcome these barriers, the Government of Bangladesh has determined that efforts to upgrade EOC facilities must continue, but complemented by a home-based strategy for skilled maternal and neonatal care (15).

Although the quality of care is an important determinant of the use of health services, it is not the only one that the provider considers while making a decision for the use of services, particularly during life-threatening morbidities. There are other equally important demand-side barriers that are not always appreciated by the provider. And these barriers are largely involved in causing delays in service recipients getting adequate care from appropriate facilities (16). Therefore, unless these barriers are carefully studied and systematically overcome, it will be difficult to achieve the Millennium Development Goals (MDGs) in general and MDG 5 in particular.

This study explored the delays in making decisions and reaching hospitals (first and second delays of the ‘three-delay model’) and the related factors for all types of illness episodes perceived to be serious, and their possible implications for safe motherhood interventions in Bangladesh.

## MATERIALS AND METHODS

Data for the study were derived from the baseline cross-sectional survey of the ‘Matlab EOC Project’ whose goal was to increase of delivery at facility and to reduce delay in accessing care for obstetric complications. The survey was conducted during December 2000–February 2001 to provide baseline information to allow evaluation of proposed safe motherhood interventions. Consequently, the study population was sampled from Matlab upazila (sub-district), which is a rural area of Bangladesh with a population of about 550,000. A two-stage World Health Organization (WHO)-standardized EPI (Expanded Programme on Immunization) 30 cluster-sampling procedure was used for identifying the study population. Villages were the primary sampling units, while households were the secondary sampling units. Villages were selected with probability proportional-to-population size, while households in selected villages were selected and located using a systematic sampling procedure by randomly selecting the first household from the first sampling interval. The household head was taken to be the respondent and, in his absence, the most informed and available adult member of the household, commonly the wife, was interviewed.

Trained interviewers located and interviewed household heads using structured questionnaires. The questions in this study focused on health-seeking behaviour in general as related to the last episode of serious illness (as perceived by the household head or his proxy) of any family member during the previous one year and decision at various stages to seek care for the sick household member. In particular, they were asked whether they perceived a delay between the onset of illness and the time they decided to seek care. Questions also focused on mode of transportation and distance to the facility or provider where care was sought. Information was also obtained about perceived delays in obtaining transport and in reaching the facility or provider. Field supervisors and research investigators closely supervised data-collection activities to ensure a high degree of data quality.

Data were entered in the Fox-Pro database-management programme and analyzed in SPSS (WIN version 10). Using the principle components and factor-analysis method, a composite socioeconomic status indicator (wealth index) was created using information on household assets, land possession, construction materials for the main dwelling house, source of drinking-water, type of latrine possessed, cash income and expenditure, and education of respondent (17–19). Bivariate and multivariate statistical tests were performed where appropriate.

## RESULTS

### Sociodemographic characteristics

In total, 2,177 households were reached, and one adult respondent from each household was interviewed. Of these respondents, 638 (29.3%) were male and 1,539 (70.7%) were female. The lower percentage of males is a reflection of daytime gender composition of households in rural Bangladesh when most men are in the field for agricultural work. The large majority of the respondents were Muslims (89.9%), while the rest were Hindus. Of the male respondents, 80.7% were household heads, while 66.0% of the females were spouses of household heads. The mean age of respondents was 38.6 years, while it was 45.1 years for males and 35.9 years for females.

Of the total number of households studied, 881 (40.5%) reported at least one member who had experienced an episode of illness perceived to be serious enough to warrant seeking care from health facilities or informal providers. Of these households, 88.0% (775 of 881) reported having actually taken sick household members to some facilities or providers for treatment after a decision had been made to do so in the household. The remaining 12% either received treatment at home or received no treatment at all. Since the people reported the last episode of disease and/or their symptoms within one year according to their own perceptions and since we wanted to capture their perceptions of the process of care-seeking and how these might have been influenced by the nature of diseases based on the ‘medical model’, we classified the reported symptoms of illnesses into three groups of presumptive medical conditions. The three groups were: (a) those likely to have been of a sudden onset and appeared acute in nature, like fever, diarrhoea, convulsions, breathlessness, trauma, etc.; (b) those likely to have been of insidious onset and appeared chronic in nature, e.g. skin disease, jaundice, kidney disease, rheumatoid arthritis, etc.; and (c) those which appeared to be related to pregnancy, childbirth and their complications.

### Perceived delay in deciding to seek care (first delay)

Responses regarding decisions to seek care for a sick household member revealed that the average time from onset of illness to the time a decision was taken to seek care was a median of 72 minutes (Table 1). In this paper, the median was used as a measure of central tendency for time and distance due to the skewness of their distributions as observed in the data. The table also shows that the proportion of the respondents perceiving a delay, classified as the first delay, was statistically significantly higher (69.3%) than that classified as the second delay (12.1% for accessing transport and 24.6% for reaching a facility). The median time taken in deciding to seek care among those who finally sought care from the nearby Health and Family Welfare Centres (HFWCs) was 168 minutes and that for seeking care from organized private facilities was 156 minutes compared to a median time of 24 minutes when care was sought from the district hospital and of 48 minutes when care was either sought from the ICDDR,B treatment centres or from the government UHC at the sub-district level.

**Table 1.** Health-seeking behaviour, socioeconomic characteristics, and perceptions about delays in seeking care

Health-seeking behaviour and socioeconomic characteristics	Median distance covered (km)	Percent using transport	Median time (minutes) taken to			Percent perceiving delay in		
			make decision	obtain transport	reach facility	making decision	obtaining transport	reaching facility
Place of treatment	(n=775)	(n=775)	(n=655)	(n=423)	(n=625)	(n=775)	(n=577)	(n=765)
Informal sector	1	52	72	10	30	62.4	13.4	16.8
HFWC	1	23.1	168	15	35	69.2	25	30.8
ICDDR,B centre	2	72	48	6	40	59.1	12.3	19.8
Upazila Health Complex	3.8	93.8	48	10	60	59.4	6.9	26.6
District hospital	20	100	24	10	180	60.0	14.3	20
Organized private sector	16	92.4	156	10	150	79.5	12	25.1
Specialized tertiary	40	98.4	96	10	300	68.9	14.3	52.5
Total	4	79.1	72	10	80	69.3	12.1	24.6
Wealth index	(n=775)	(n=775)	(n=655)	(n=423)	(n=625)	(n=775)	(n=577)	(n=765)
Poorest quintile	2	66.2	72	10	60	69.2	17.3	22.7
Second quintile	3	75.2	72	10	60	68.2	13.1	21.1
Third quintile	3	75.5	72	10	60	66.2	13.1	24.8
Fourth quintile	8	83.3	120	10	120	73.7	10.4	25.8
Richest quintile	15	90.2	96	7	120	68.9	9.7	26.7
Total	4	79.1	72	10	80	69.3	12.1	24.6
Sex of patient	(n=775)	(n=775)	(n=655)	(n=423)	((n=625)	(n=775)	(n=577)	(n=765)
Male	4	80	72	10	60	65.8	12.3	24.5
Female	4	78.2	96	10	90	72.7	11.9	24.7
Total	4	79.1	72	10	80	69.3	12.1	24.6
Admission status	(n=775)	(n=775)	(n=655)	(n=422)	(n=624)	(=774)	(n=576)	(n=764)
Admitted to hospital	10	95.3	48	10	90	60.0	14.6	27.1
Not admitted to hospital	3	74.5	96	10	70	71.9	11.2	23.9
Total	4	79.1	72	10	80	69.3	12.2	24.6

HFWCs=Health and Family Welfare Centres;

ICDDR,B=International Centre for Diarrhoeal Disease Research, Bangladesh

Table 1 also shows that the median distance covered to reach treatment facilities was four km, being shortest (1 km) for patients accessing care from informal providers or the HFWCs and those from the poorest quintile households, and longest for patients attending treatment in specialized tertiary facilities and those from the richest quintile households. The table also summarizes information about the percentage using transport to facilities (79.1%), the median time to obtain transport (10 minutes), the proportion perceiving delay in getting transport (12.1%), the median time to reach facility (80 minutes), and the proportion perceiving delay to reach a facility (24.6%). Patients from the richest quintile households covered the longest distance (median of 15 km) and consisted of the highest proportion using transport (90.2%) and the lowest proportion using the services of informal providers (14.5%), while they took the longest time to reach a facility. However, there was no significant difference among the socioeconomic groups in perceptions of delay in deciding to seek care (p=0.14), in reaching facility (p=0.10), or in getting transport (p=0.59). Likewise, gender perceptions of delay did not vary significantly regarding decisions to seek care, getting transport, or reaching facility. However, a significantly higher proportion (79.5%) of those seeking care from organized sector facilities perceived a delay in deciding to seek care than the proportion (62.4%) of those seeking care from informal providers.

**Fig. 1.**
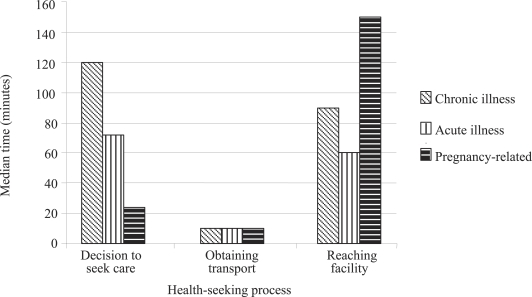
Perceived delay in health-seeking according to type of illness

Figure 1 shows an analysis of type of illness and the health-seeking process. The figure shows that it took the shortest time (24 minutes) to take a decision about seeking care for pregnancy-related conditions, while it took the longest time (120 minutes) for conditions classified as chronic. The time to obtain transport was shown to be the same (10 minutes) regardless of type of illness. However, the reported time taken to reach a facility was longest (median of 150 minutes) for pregnancy-related conditions and shortest (60 minutes) for conditions classified as acute, implying that most women with pregnancy-related complications sought care from distant facilities (53% from the organized private sector, 13% from the district hospitals, and 13% from the tertiary-level referral facilities), which, in turn, may have influenced the shorter time they took to decide to seek care compared to other types of illness. Only 22 women were reported to have suffered from serious pregnancy-related illnesses compared to 530 patients in the chronic category and 329 patients in the acute category.

### Perceived delay in accessing transport and in reaching selected facility (second delay)

Of those who visited a health facility or an informal provider, 79.1% used some form of transport to reach the selected facility or provider. Of those reporting transport use, 56.6% used three-wheeled rickshaws and rickshaw vans, while 32.9% used engine-propelled boats. In addition, 30.2% used motorized road transport, and 13.9% used ordinary country boats, while only 0.8% used hospital ambulances. The above percentages add up to more than 100% because some patients used more than one means of transport to reach a chosen facility or provider.

The proportion reporting transport use increased with distance to the selected health facility only up to the first five km, beyond which reported transport use was almost universal. Only 23.1% of patients seeking care from the HFWCs and 52.0% of those seeking care from the informal private sector required transport, while 98.4% (60 of 61) of those seeking care from the specialized tertiary facilities used transport, a finding that is most likely due to the greater distance of specialized facilities from the patient's home. Notably, transport was used by 100% of those seeking care from the government district hospital. The use of transport to reach facilities was reported by a higher proportion of patients coming from the richest quintile households (90.2%) than that from the poorest (66.2%) and was also highest among those reporting illnesses classified as chronic (82.4%) and lowest among those reporting acute forms of illness (74.0%), the differences being statistically significant (p<0.0001 and p=0.02 respectively).

When the mean times were compared between those who perceived a delay and those who did not, the mean time among those perceiving a delay was significantly higher than that of those not perceiving a delay, indicating that the perceived delay can be regarded as a good indicator/proxy for actual delay since no actual measurements of time or distance were made in this study.

Table 2 shows the reasons for delay in seeking care according to socioeconomic status. The main reason given for delay in seeking care was that the patient was already under treatment by informal providers (39.9%), the lowest percentage being in the poorest quintile households (28.9%). Another important reason mentioned was the inability to judge the graveness of illness, which was given by 29.4% of those who sought care. Lack of money for transport and treatment was mentioned by 21.0% of the respondents as a reason for delay in deciding to seek care, the highest percentage being in the poorest quintile (37.8%) and the lowest in the richest quintile (9.8%). The table also shows the reasons for delay in reaching a facility, which included delay by the slow speed of the transport means and delay by going on foot for lack of transport or money.

**Table 2.** Reasons for delay in deciding to seek care and in reaching facility by economic status

Reason for delay	Socioeconomic status											
	Poorest quintile		2^nd^ quintile		3^rd^ quintile		4^th^ quintile		Richest quintile		Total	
	No.	%	No.	%	No.	%	No.	%	No.	%	No.	%
Delay in deciding to seek care												
Already under informal care	26	28.9	45	42.1	32	34.8	54	42.9	57	42.9	214	39.9
Unable to judge graveness of illness	24	26.7	29	27.1	27	29.3	29	25.2	49	36.8	158	29.4
Not having enough money	34	37.8	23	21.5	21	22.8	22	19.1	13	9.8	113	21.0
No appropriate escort	3	3.3	6	5.6	6	6.5	6	5.2	7	5.3	28	5.2
Other reasons	3	3.3	4	3.7	6	6.5	4	3.5	7	5.3	24	4.5
Total	90	100	107	100	92	100	115	100	122	100	537	100
Delay in reaching hospital												
Slow speed of vehicle	9	3.1	13	38.2	18	52.9	18	46.2	28	56.0	86	46.2
Went on foot for lack of transport	13	44.8	9	26.5	6	17.6	6	15.4	1	2.0	35	18.8
Frequent stoppage	3	10.3	4	11.8	5	14.7	6	15.4	7	14.0	25	13.4
Busy road/traffic jam	0	0.0	4	11.8	3	8.8	9	23.1	11	22.0	27	14.5
Other reasons	4	13.8	4	11.8	2	5.9	0	0.0	3	6.0	13	7.0
Total	29	100	34	100	34	100	39	100	50	100	186	100

**Fig. 2.**
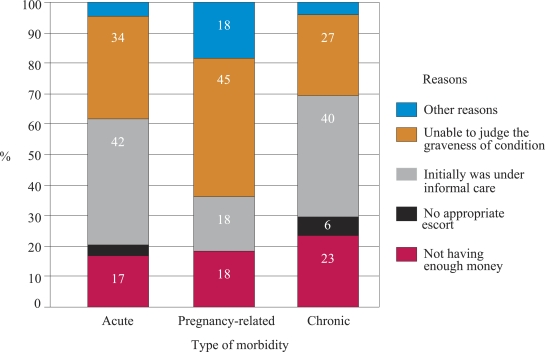
Reasons for delay in making decision for seeking care by type of morbidity faced

Figure 2 shows that the main reason for delay in decision-making to transfer the patient by type of disease suffered. For pregnancy-related morbidities, 45% mentioned ‘inability to judge the graveness of the situation during appearance of initial symptoms’ as the main reason for delay in making a decision to seek care.

Since the first delay was the most important barrier to accessing care and due to the possible influence of extraneous factors, such as distance to facility, socioeconomic status, place of treatment, sex of patient, and religion, on the decision to seek care, a multivariate analysis was performed to control for their influences. It was found that the type of illness and the facility chosen were the most important determinants of delay in reaching a decision to seek care. Hence, for any episode of illness requiring facility care, time to decide to seek care was determined by type of facility chosen for treatment.

## DISCUSSION

The three-delay model of accessing care is an important concept in safe motherhood. This baseline study attempted to capture the health-seeking behaviour of the community people for the last episode of perceived severe morbidities faced by any family member before upgrading the peripheral government facilities to provide basic and comprehensive EOC services. It focused only on the first and the second delay of the three-delay model since the proposed facilities where the third delay could be studied had not been completed. The findings indicate that delay one, i.e. the time taken to make a decision to seek care was prolonged and that was well-perceived by the majority of the community people. Getting transport was not a problem in rural Bangladesh when a decision to seek care was made, but reaching facilities took a longer time particularly when treatment was sought from higher levels of healthcare-delivery systems. The proportions of those perceiving delay in deciding to seek care were significantly higher than of those perceiving delay in reaching a facility in all illness categories, indicating that the most important factor contributing to delay in accessing care for any episode of illness in rural Bangladesh is a delayed decision to seek care. The Bangladesh Maternal Health and Maternal Mortality Survey (BMMS) 2001 showed a similar picture where household took 2.8 hours (median) to recognize a life-threatening obstetric complication and the median delay between recognizing complications and deciding to seek care was 1.6 hours (7). Results of the BMMS 2001 also showed that those who sought treatment for life-threatening complications, three-fourths of women could reach a facility within one hour and one-third within half an hour, suggests that accessing facilities is not a problem in Bangladesh, the problem is the delayed decision-making process for seeking care.

During the course of any episode of illness, the patient uses several strategies to deal with symptoms that include self-treating, ignoring, positive thinking, and waiting or being under treatment of informal providers at home, while recourse to a professional is used as the strategy of last resort when symptoms can no longer be contained (20). In our study, most of these strategies may have, we believe, played an important role in delaying the process of seeking care, although they may not have been reported (21), and much time is taken in consultations and dialogue by those concerned, thereby constituting possible delays in making decisions for seeking care. Once a decision is made about care-seeking, patients tend to use more than one provider in the course of any one given episode of illness (22). All in all, such patient strategies are common and complex and often contribute to the delay to seek care, a situation that may have influenced our findings. Another limitation is that morbidities are not clinically-diagnosed medical conditions rather perceived serious problems reported by the household head or his proxy.

In our study, inability of the respondents to recognize the graveness of illness condition, lack of money, and use of informal providers were important reasons given for delays. Other studies have observed similar findings (23). The findings of our study revealed that, for pregnancy-related morbidities, the most commonly-cited reason for delay in making a decision to seek care was ‘inability to judge the graveness of the situation’. It implies that there is a need to educate the community people on ‘pregnancy-danger signs’ so that they can recognize symptoms early and can make decisions promptly to transfer the patient to appropriate level of care. Such findings also call for intensified efforts to encourage them to be financially prepared for possible emergencies to transfer patients to relevant health facilities for appropriate care. However, that is not possible without an active programme to raise awareness about these so that they can recognize these pregnancy-danger signs on time to take necessary actions towards treatment. Similarly, households required more time to reach facilities when it was a pregnancy-related morbidity, which indicates that decentralization of EmOC facilities had been the right step to improve the maternal health scenario of the country. Accessing facilities on foot was another important reason for delay in reaching facilities among the poorest group. These reasons imply that lack of money is an important determinant of the use of health services as has been shown by a recent study in Bangladesh that compared users and non-users of EOC facilities and found that economic status seriously influenced people's decision to use EOC facilities (24). Our study focused on users of services and has shown that perceptions of delays to seek care had a similar pattern across all socioeconomic groups, indicating that the need for healthcare is inelastic and does not depend on the economic status. Simultaneously, the poorest families mostly cited ‘not having enough money’ as the main reason for delay in making a decision to seek care. Hence, the demand-side financing programmes, recently adopted by the Government, look promising in overcoming the access barriers for the extreme poor and, thus, contribute to achieving the MDG goals.

In the field of safe motherhood, making appropriate EOC services available close to the community and raising awareness about these have been shown to have a significant influence on health-seeking behaviour and pregnancy outcomes (25). Education about pregnancy-danger signs and about appropriate EOC services should target women and those who may influence their decisions to seek care to reduce unwarranted delay and to allow them to make informed choices about the use of healthcare. The findings of our study showed that transport is necessary beyond five km of distance. The HFWCs were the closest facilities to the households, and since these are the facilities, which the Government is planning to upgrade to provide basic EOC services, requirement of transport to reach these facilities was minimal. Time to obtain transport was short for all categories of illness episodes. Although it took more than one hour for the majority of the patients to reach a facility (median of 80 minutes), only a small proportion (25%) of the respondents perceived this as a delay, implying that, for the majority of the patients, the time taken to reach a facility was the minimum possible, given the means of transport taken and the distance covered. It implies that bringing services closer to communities can significantly shorten the time to reach facilities and, hence, improve their use. These findings reflect those of others suggesting improvements in the availability of transport for remote areas (26) and use of district and sub-district-level facilities that are equitably distributed and upgraded to treat obstetric complications (27, 28). This strategy is supported by a recent study which suggests that EOC facilities in Bangladesh should be close enough to minimize social costs and to increase use (29). Thus, the efforts of the Government of Bangladesh to upgrade UHCs to provide comprehensive EOC services and basic EOC services at the HFWCs constitute a major step towards that direction.

In Sudan, results of a study showed that, generally, women did not wish to spend more than 30 minutes in reaching a facility, but, if the quality of a particular institution was considered to be good and supplies and equipment were available, women would cover great distances to reach such a facility (30). Results of studies in Guatemala and India showed that the low use of government facilities was mainly due to the poor quality of services that motivated women to deliver at home rather than the influence of physical access or cultural barriers to facility use (31, 32). Other studies have shown that mismanagement of services and corruption may increase the cost of seeking care, which may, in turn, act as a deterrent to timely decision-making by households to seek care (33). Findings of these studies indicate that policy-planners should first ensure the presence of quality services in facilities that are affordable and close to communities. There must be continuous efforts to improve and sustain the quality of care in these EOC facilities. Thus, the process of upgrading government healthcare facilities, training of community-based skilled birth attendants (SBAs), and introduction of a voucher programme must go hand in hand with creation of demand by proving behavioural change communication (BCC), particularly on life-threatening obstetric complications, and a quality-improvement initiative. Recently-trained community-based SBAs might be the best vehicle to impart BCC for women and other potential decision-makers in the community.

If the perceptions of delay in deciding to seek care and to reach a health facility for general illness as observed in this study can be translated into reality, their implications for safe motherhood in countries like Bangladesh should be tremendous. Hence, appropriate BCC for communities to recognize pregnancy-danger signs should be initiated to enable women, communities and their gatekeepers to reduce delays to seek care. This is particularly so for life-threatening conditions, such as antepartum haemorrhage, postpartum haemorrhage, and eclampsia.

However, to educate communities to change and make timely decisions regarding care-seeking will take time, and it will, therefore, be necessary to develop strategies in line with ongoing maternal and neonatal health interventions. The EmOC strategy is now supplemented by the home-based SBA strategy (34), and a voucher scheme is being piloted in many sub-districts of the country. And the interventions are placed in such a manner that will allow the research community to initiate a ‘natural experiment’ in the field of maternal and neonatal health. To that end, the findings of our study suggest that there is a need for research examining the feasibility and testing the effects of imparting BCC with the newly-trained community-based SBAs in Bangladesh who, in addition to providing skilled attendance at home, can influence the community to be aware of pregnancy-danger signs and availability of maternal and neonatal healthcare services in the area. These community-based SBAs, if equipped with appropriate BCC packages, will be able to influence decisions to seek care at the community level during the antenatal, natal and postnatal periods by helping to identify obstetric complications for early referral and, hence, reduce the delay in accessing appropriate care. By combining this community-based SBA strategy with the current improvements in the upgrading of facilities close to the communities, it should be possible to shorten the first, second and third delays considerably, thereby reaching the safe motherhood target of reducing maternal mortality sooner as targeted in MDG 5.

In our study, the first delay was found to be the most important barrier for accessing care for episodes of illness perceived as serious. Failure to recognize the graveness of illness and lack of money for transport were important reasons for delay in seeking care. To reduce delays to seek care and to improve access to EmOC in rural Bangladesh, interventions should be intensified to enable communities to recognize ‘pregnancy-danger signs’ for which action must be taken to save life and to save money for emergency needs of transport and for cost of treatment. This strategy should be combined with ongoing community-based SBA efforts and decentralization of EmOC and to improve the quality of care in the EOC service-delivery systems.

## ACKNOWLEDGEMENTS

The study was conducted at ICDDR,B with support of a grant from the Commission of the European Communities (grant no. ALA/97/0038 and contract no. BGD/B7-300/0038-01). ICDDR,B acknowledges with gratitude the commitment of the Commission of the European Communities to the Centre's research efforts.
